# Mapping the landscape and structure of global research on nutrition and COVID-19: visualization analysis

**DOI:** 10.1186/s41043-022-00304-y

**Published:** 2022-06-10

**Authors:** Sa’ed H. Zyoud, Samah W. Al-Jabi, Amer Koni, Muna Shakhshir, Moyad Shahwan, Ammar A. Jairoun

**Affiliations:** 1 Poison Control and Drug Information Center (PCDIC), College of Medicine and Health Sciences, An-Najah National University, Nablus, 44839 Palestine; 2 Department of Clinical and Community Pharmacy, College of Medicine and Health Sciences, An-Najah National University, Nablus, 44839 Palestine; 3 Clinical Research Centre, An-Najah National University Hospital, Nablus, 44839 Palestine; 4 Division of Clinical Pharmacy, Hematology and Oncology Pharmacy Department, An-Najah National University Hospital, Nablus, 44839 Palestine; 5 Department of Nutrition, An-Najah National University Hospital, Nablus, 44839 Palestine; 6 College of Pharmacy and Health Sciences, Ajman University, Ajman, United Arab Emirates; 7 Health and Safety Department, Dubai Municipality, Dubai, United Arab Emirates

**Keywords:** Nutrition, COVID-19, Scopus, VOSviewer

## Abstract

**Background:**

Coronavirus disease 2019 (COVID-19) has had a significant influence on nutritional status. There have been several studies on dietary habits and nutritional status in connection with COVID-19. However, there has been no research on the bibliometric analysis of these papers. Therefore, our objective was to assess the most relevant scientific research on nutrition and COVID-19, as well as to assess current hot themes.

**Methods:**

We obtained data from the Scopus database on June 30, 2021. Qualitative and quantitative analyzes were conducted based on the Scopus. Collaboration and term analysis was performed using VOSviewer software version 1.6.16.

**Results:**

At the time of data collection, there were 177,946 documents in COVID-19. Scopus found 1885 articles related to nutrition and COVID-19 after narrowing the search to those terms. This includes 1309 (69.44%) research articles, 268 (14.22%) review papers, and 308 other types of document. The USA was the largest producer, with 24.83% of the documents, followed by Italy with 11.88% (*n* = 224), the UK with 10.82% (*n* = 204), and China with 7.59% (*n* = 143). The most active institution was *Sapienza Università di Roma* (*n* = 30, 1.59%). The leading journal in COVID-19 nutrition research was *Nutrients *(*n* = 115, 6.10%). The article with 310 citations published by Di Renzo et al*.* in 2020 was the most influential reference. The hot topics were stratified into three clusters: (1) “Food security in the COVID-19 pandemic”; (2) “nutritional determinants and COVID-19 outcomes”; and (3) “changes in dietary habits during the COVID-19 pandemic and its consequences”.

**Conclusions:**

This is the first bibliometric research to offer comprehensive information on COVID-19 and nutrition in the published literature. Research will likely be helpful to scholars and policymakers. This study sheds light on the growth and development of nutrition and covid-19-related research and should contribute to the expansion of the global frontier in the major hot topics, including “food security in the COVID-19 pandemic”; “nutritional determinants and COVID-19 outcomes”; and “changes in diet habits during the COVID-19 pandemic and its consequences”.

## Background

The threat of coronavirus disease 2019 (COVID-19) to global security and economy has captured the attention of the entire world [1]. COVID-19 has been causing havoc in the global system at various levels and changing the lifestyles of the public [2]. Moreover, countries with a low or middle income complain of nutritional deficiency, which is expected to worsen during the COVID-19 crisis [3]. Therefore, this unprecedented pandemic has many undesirable influences, not only on the food supply chain [4] but also on food security, including nutritional status and diet habits [5]. As mentioned recently, global food systems are desperately in desperate need of a sustainable and nutritional overhaul [6], as evidenced by the fact that food and nutrition are linked to all Sustainable Development Goals (SDGs) of the United Nations Sustainable Development Goals (SDGs) [7].

Previous reports linked the consumption of an unhealthy diet with the risk of mortality from noncommunicable diseases [8, 9]. However, the impact of bad eating habits is not limited to that, but may extend to infectious diseases such as COVID-19 [10]. Many articles focused on the benefits of consuming diets rich in antioxidants, such as essential vitamins and minerals, as well as a commitment to the Mediterranean diet, and at the same time avoiding refined foods and saturated lipids wherever possible, which are the composition of the Western diet, improving immune functions and subsequently, decreasing the risk of COVID-19 and severe events [10–13].

Population eating behaviors have been changing throughout the world, particularly those in quarantine due to the COVID-19 crisis [2, 14]. It should be noted that a study showed that people tend to consume unhealthy nutrition and decrease bodily activities during lockdown [2]. Another study found that people whose body mass index was ≥ 25 were more likely to have unhealthy eating habits [15]. On the other hand, the COVID-19 crisis has profound effects on food security, in which it can weaken the ability of people to get essential nutrition due to stock shortages or food costs [5, 16]. Furthermore, some research provided certain recommendations and tactics to the public to maintain and improve their nutritional status during the COVID-19 pandemic [12, 17].

Most prior research examined the worldwide scientific literature on COVID-19-related topics, such as COVID-19 and older adults [18], COVID-19 pandemic and sustainable development goals [19], rheumatology and COVID-19 [20], COVID-19 and depressive disorders [21], COVID-19 and ophthalmology [22], dental scientific literature on COVID-19 [23], COVID-19 and diabetes [24], and COVID-19 in environmental [25], using bibliometric network analysis. However, according to the literature review, scientific trends and hotspots in relation to the pandemic and its association with nutrition are unknown. Bibliometric is a useful technique for quantifying and qualitatively analyze changes in research activity over time. It has grown in popularity to obtain knowledge in particular subjects [26], and it is important to guide future research priorities. Therefore, our objective was to assess the most relevant scientific research on nutrition and COVID-19, as well as to assess current hot themes.

## Methods

### Data source

Scopus' online database was used to search for published publications (last access date: June 30, 2021). Scopus is an Elsevier-owned abstracting and citation database and includes the most relevant scientific search engines and databases for retrieving bibliometric data. Compared to other databases such as PubMed, Web of Science, and Google Scholar, Scopus is widely considered the primary quality-oriented database on a global scale, containing a more standardized record for retrieving the global scientific literature in a variety of research areas [27, 28].

### Search strategy

We used an advanced search in the Scopus database to find relevant publications on nutrition and COVID-19 during the early stages between January 1, 2020 and June 30, 2021. To avoid the risk of bias induced by constant database changes, the retrieval and export of articles should occur within a single day (June 30, 2020). The data from this study were recovered using the following strategy:

- Step 1: Reviewing the literature (i.e., systematic reviews and meta-analysis studies) on nutrition and COVID-19 research to identify relevant keywords for search.
- Step 2: To achieve the objectives of the terms linked with COVID-19 were put into the Scopus research engine. They were derived from earlier COVID-19 bibliometric studies [29–37]. All chosen "terms" were put in the section "Article Title/Abstract/Keywords."
- Step 3: Subsequently, we limited the documents identified in step 2 to those having the phrase "nutrition and associated terms" in their titles. Nutrition-related terms were extracted from PubMed Medical Subject Headings (MeSH) and from prior systematic and meta-analysis with COVID-19 in the nutrition field [38–41] and entered into the Scopus Engine.

### Bibliometric indicators

This study assessed the following areas of research: (1) publication output and article types; (2) the top ten most influential journals with their impact factors; (3) the top ten most cited articles; (4) the top ten countries' research productivity; and (5) the top ten institutes' research productivity.

### Data analysis and visualization

Social network analysis was used to identify dynamic patterns of emphasis and links between the top producing countries and hot topics related to the investigated topic. This analysis was carried out in this study using the user-friendly program VOSviewer version 1.6.16 (created by Leiden University in the Netherlands). VOSviewer assists in creating clear information for visualization in many knowledge fields by displaying a graphic map of the bibliometric data's relationships in the cluster [42, 43].

## Results

### Volume of publications

At the time of data collection, there were 177,946 documents in COVID-19 (June 30, 2021). Scopus found 1885 articles after narrowing the search to those related to nutrition and COVID-19. This includes 1309 (69.44%) research articles, 268 (14.22%) review papers, and 308 as other types of documents.

### Contributions of countries to global publications

**Fig. 1:**
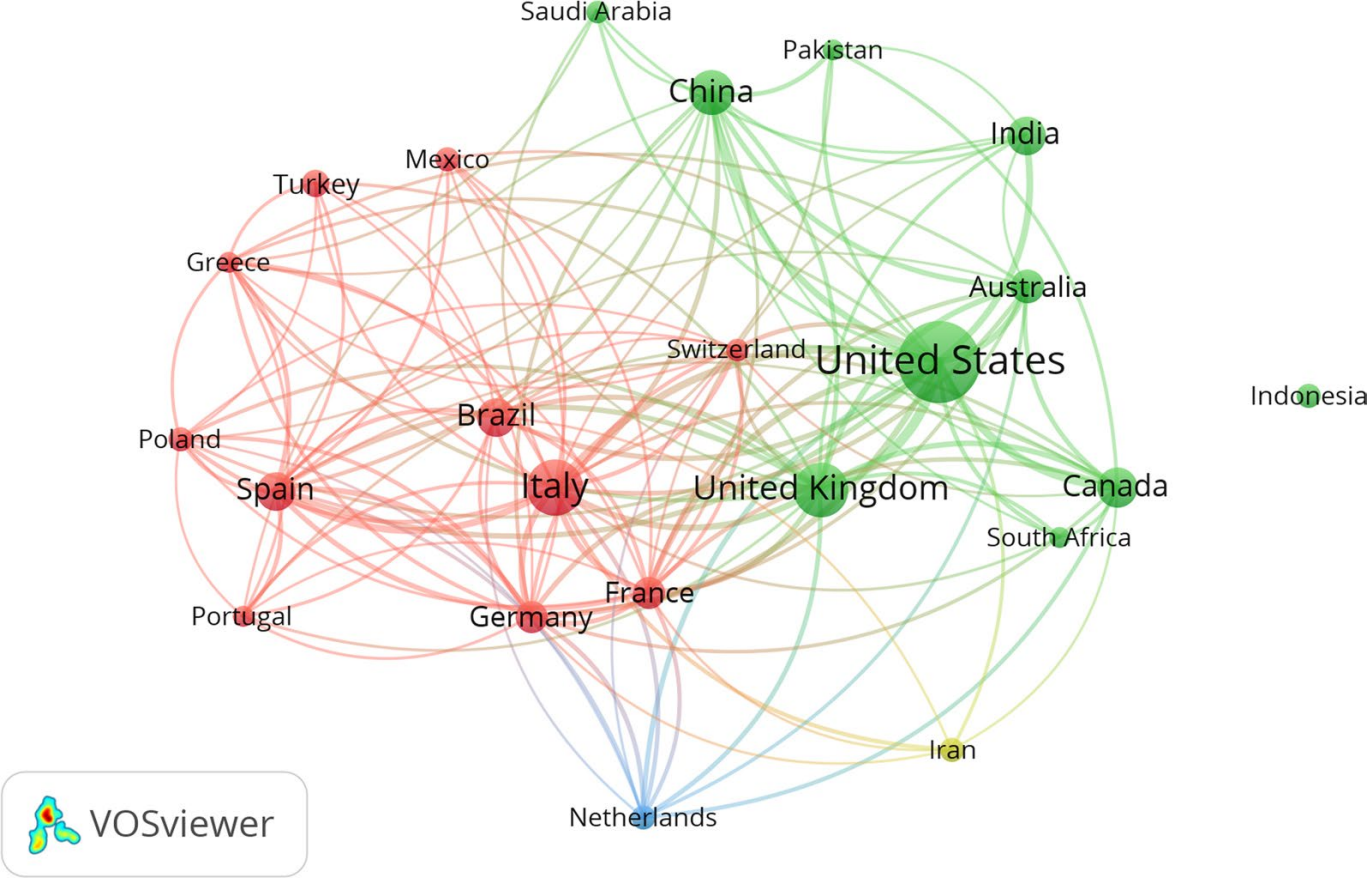
Shows the collaborative network of the most productive countries. This graphical collaboration map was generated after at least 30 publications were placed in each country. This criterium was met by 23 countries out of 167 countries published in this field. The size of the node represents the number of publications for that country

A total of 167 countries contributed to the production of nutrition and COVID-19 research. The top ten most productive countries are presented in Table 1. This analysis shows that ten countries represent 85.20% of production, with the USA being the largest producer, with 24.83% of the documents, followed by Italy with 11.88% (*n* = 224), the UK with 10.82% (*n* = 204) and China with 7.59% (*n* = 143). As seen in Fig. 1, a network of countries was constructed. This network has 23 nodes, each of which represents a distinct country with a minimum of 30 articles, and each link indicates a relationship between two countries. The USA, followed by Italy, the UK, and China, are at the forefront of collaboration and have the strongest alliance ties in research with other nations.

**Table 1:** Top ten countries published in the field of nutrition and COVID-19

Ranking	Country	Number of documents	%
1st	USA	468	24.83
2nd	Italy	224	11.88
3rd	UK	204	10.82
4th	China	143	7.59
5th	Canada	109	5.78
6th	India	106	5.62
7th	Spain	101	5.36
8th	Brazil	100	5.31
9th	Australia	78	4.14
10th	Germany	73	3.87

### Active institutions/organizations

Table 2 shows the top ten active institutions for nutrition and COVID-19 related articles. *Sapienza Università di Roma* (*n* = 30, 1.59%) was the top, followed by *the International Institute of Food Policy Research* (*n* = 25, 1.33%) and *Università degli Studi di Milano* (*n* = 24; 1.27%). The majority of active institutions were from Italy (*n* = 3), followed by the USA (*n* = 2), France (*n* = 2), and one institute from Canada, the UK, Saudi Arabia and Brazil.

**Table 2:** Top ten organizations published in the field of nutrition and COVID-19

Ranking	Country	Country	Number of documents	%
1st	*Sapienza Università di Roma*	Italy	30	1.59
2nd	*International Food Policy Research Institute*	USA	25	1.33
3rd	*Università degli Studi di Milano*	Italy	24	1.27
4th	*Harvard Medical School*	USA	17	0.90
4th	*INRAE*	France	17	0.90
6th	*King Saud University*	Saudi Arabia	16	0.85
7th	*Inserm*	France	15	0.80
7th	*Universidade de Sao Paulo - USP*	Brazil	15	0.80
7th	*King's College London*	UK	15	0.80
7th	*University of Toronto*	Canada	15	0.80
7th	*Università degli Studi di Napoli Federico II*	Italy	15	0.80

### Active journals

The top ten journals are *Nutrients* (*n* = 115, 6.10%), *Sustainability Switzerland* (*n* = 52, 2.76%), *International Journal of Environmental Research and Public Health* (*n* = 46, 2.44%), *Appetite* (*n* = 35, 1.86%), *Public Health Nutrition* (*n* = 31, 1.64%) and so on (Table 3).

**Table 3:** List of the top ten productive journals in the field of nutrition and COVID-19

Ranking	Journal	*n*	%	IF^a^
1st	*Nutrients*	115	6.10	5.717
2nd	*Sustainability*	52	2.76	3.251
3rd	*International Journal of Environmental Research and Public Health*	46	2.44	3.390
4th	*Appetite*	35	1.86	3.868
5th	*Public Health Nutrition*	31	1.64	4.022
6th	*Food Security*	29	1.54	3.304
7th	*Clinical Nutrition*	28	1.49	7.324
7th	*Frontiers in Nutrition*	28	1.49	6.576
9th	*Clinical Nutrition Espen*	21	1.11	NA
10th	*Trends in Food Science and Technology*	20	1.06	12.563

*IF* impact factor, *NA* not available

^a^Journal citation reports (Source Clarivate, 2021)

### Top-cited publications

The top ten articles were cited 2012 times in total number of, and the average total citations was 182.9 (ranging from 116 to 310) [2, 5, 10–12, 14, 15, 17, 44, 45]. Table 4 shows the top ten publications with more than 166 citations.

**Table 4:** The top ten articles with most total citations in the field of nutrition and COVID-19

Ranking	Authors	Year	Source title	Cited by
1st	Di Renzo et al. [14]	2020	*Journal of Translational Medicine*	310
2nd	Ammar et al. [2]	2020	*Nutrients*	285
3rd	Barazzoni et al. [44]	2020	*Clinical Nutrition*	223
4th	Calder et al. [11]	2020	*Nutrients*	201
5th	Galanakis [5]	2020	*Foods*	180
6th	Hobbs [45]	2020	*Canadian Journal of Agricultural Economics*	175
7th	Sidor and Rzymski [15]	2020	*Nutrients*	153
8th	Butler and Barrientos [10]	2020	*Brain, Behavior, and Immunity*	136
9th	Muscogiuri et al. [17]	2020	*European Journal of Clinical Nutrition*	117
10th	Zabetakis et al. [12]	2020	*Nutrients*	116

### Research themes in nutrition and COVID-19-related literature

**Fig. 2:**
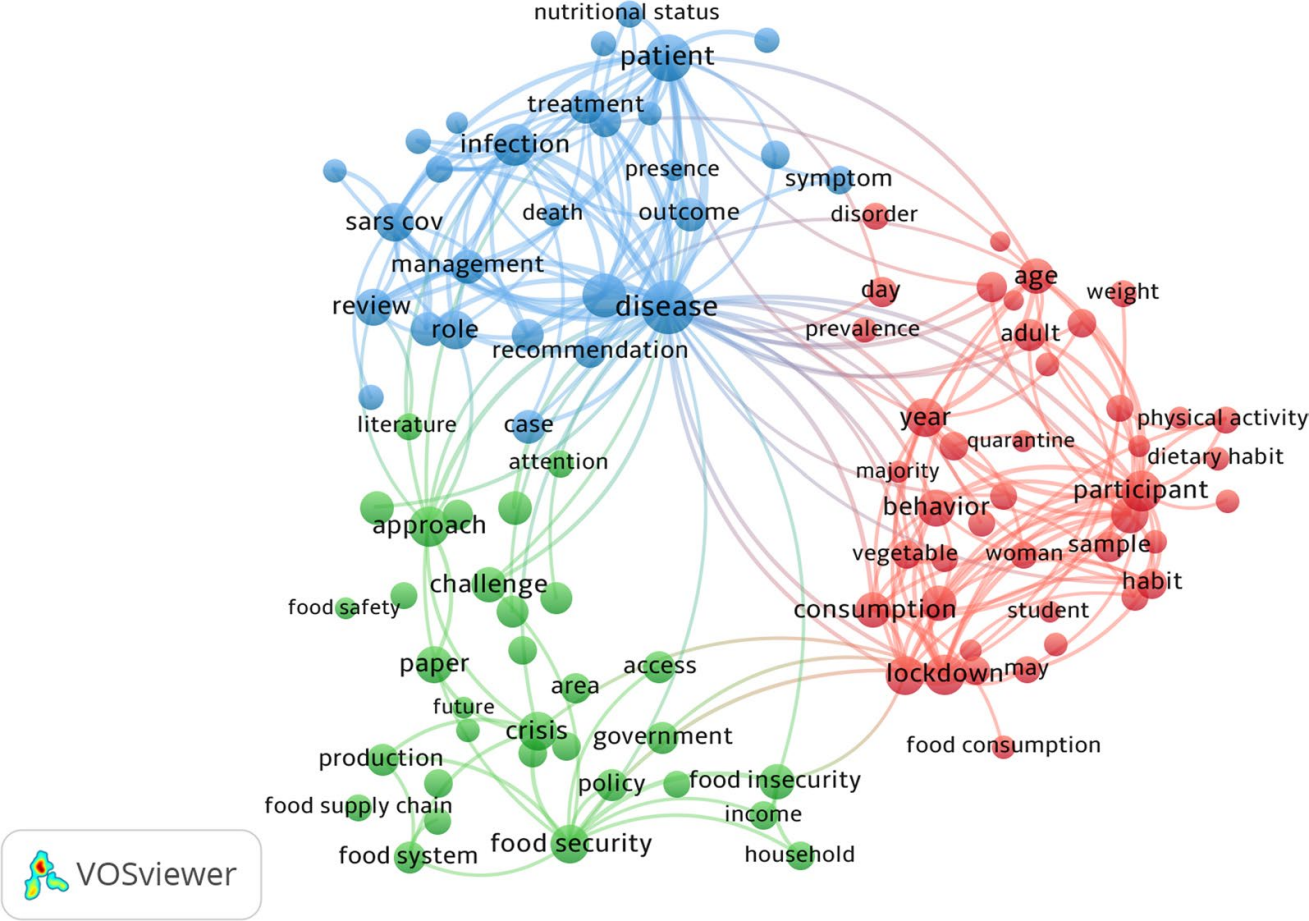
Visualization of the network of publications associated with nutrition and COVID-19 based on the titles/abstracts of the retrieved literature. Each node in the map indicates a term that appeared at least 50 times, and the size of a term's node is proportional to the number of times that the term appeared. Cluster analysis based on term cooccurrence showed three primary clusters (green, blue, and red). ‘Food security in the COVID-19 pandemic’ (cluster 1, green), ‘nutritional determinants and COVID-19 results’ (cluster 2, blue), and ‘changes in dietary habits during the COVID-19 pandemic and its consequences’ (cluster 3, red) are the three clusters

The terms most commonly used in the titles and abstracts of articles related to nutrition and COVID-19 are depicted in Fig. 2. The bigger the circle, the more frequently a given phrase appears, and the narrower the space between two terms or circles, the more frequently the terms appear together. Colors represent groups of terms that are closely related. Cluster analysis based on term cooccurrence showed three primary clusters (green, blue, and red). The key terms related to ‘changes in dietary habits during the COVID-19 pandemic and its consequences’ are located in the red group and are significantly associated with terms from the other groups. ‘Food security in the COVID-19 pandemic’ (cluster 1, green), ‘nutritional determinants and outcomes of COVID-19’ (cluster 2, blue) and “changes in dietary habits during the COVID-19 pandemic and its consequences’ (cluster 3, red) are the three clusters (Fig. 2). Food security, food insecurity, food system, food supply, food production, and food safety are the most prominent terms in cluster 1. Nutritional status, outcome, patient, death, management, symptoms, and treatment are some of the most prevalent terms in cluster 2. Dietary habits, physical activity, vegetable food consumption, and weight are the most often used terms in cluster 3.

## Discussion

Current scientific research is critical for the prevention and control, particularly during the COVID-19 pandemic that leads to a high worldwide death rate worldwide [46]. Although most of the scientific literature was created in a relatively short period of time, an examination of research related to the COVID-19 pandemic is necessary. This bibliometric analysis is unique in that it is the first to identify and evaluate the features of scientific publications on COVID-19 nutrition scientific publications released during the early stages of the pandemic. The analysis of 1885 scientific articles published between January 2020 and July 2021 reveals which countries and institutions have contributed the most publications and which journals have published the most volumes of COVID-19 nutrition research and highlights the most cited publications and the main research issues addressed.

The USA, Italy, China and the UK had the most published COVID-19 nutrition research published in the literature, accounting for 55.12% of all publications in the study. Although no bibliometric study on COVID-19 nutrition research has been published, Nemours studies have been conducted on COVID-19 research productivity in various fields [18, 20–25, 47–50], as measured by publications, and found that the USA, the UK, China and Europe were the top producers of COVID-19 publications during this time.

Three main research themes in COVID-19 nutrition research were recognized based on the analysis of terms and specified fields of research interest. This analysis found the most common terms in the scientific literature and showed how they occurred in several publications. ‘Changes in diet patterns during the COVID-19 epidemic and its consequences’ as a theme were among the main hot topics in the current study. During the pandemic, the frequency with which people consumed different types of food decreased [51]. There was also a decrease in physical activity and an increase in sedentary behavior [51]. During pandemic-induced lockdown or quarantine, social isolation had a more severe impact on nutritional status [52]. COVID-19 has had a great influence on some communities [53–56], leading them to change their dietary habits. During the COVID-19 pandemic, the influence of social isolation and lockdowns on eating patterns should not be ignored, as it has already had acute impacts and will most likely have long-term negative consequences for public health. Poor dietary habits that are continued over time will increase plasma risk factors for cardiovascular disease, diabetes, and cancer [57, 58].

Another subject that has received a lot of attention during the COVID-19 epidemic is food security. Maintaining the security of our food supply and having quick access to adequate amounts of safe food is of key importance to all people during this pandemic is one of the most significant problems for the food system. The pandemic may have had an impact on global food security [16, 59–69]. Food security is a key component of the 2030 Agenda for the Sustainable Development Goals, which seek to eradicate poverty and protect the environment [7]. Delays in food delivery, loss of food quality and quantity, limitations in food access, and income shortages to purchase food have all occurred in third-world nations. This has immediate health consequences, and given the detrimental effects of previous pandemics on human growth and health, it is reasonable to expect that the present COVID-19 pandemic will cause nutritional deficiencies around the world, with long-term consequences for human health [57]. Furthermore, COVID-19 has emphasized the need for early diagnosis of novel infectious diseases, 70% of which originate in animals. Improving the surveillance systems for zoonotic diseases resulting from animals used in the food chain is critical to preventing future disasters [16].

Another hot topic is the determinants and COVID-19 outcomes. In reviewing the literature, several articles hypothesize that a balanced diet could help reduce the prevalence of COVID-19 infection and alleviate its clinical symptoms [10, 70–74]. Nutrients such as vitamins, zinc, and fibers should be consumed to boost immunity through their antioxidant activities or anti-inflammatory effects [13]. Although the risk of infection and the severity of clinical symptoms may be influenced by the food a person eats, it is not possible to completely avoid viral transmission by maintaining a healthy diet or using dietary supplements. More studies are needed to understand the effect of vitamin D and zinc levels in patients with COVID-19 patients on viral transmission and their clinical symptoms [57].

Citation analysis is one of the most significant methods for determining the effect of an article's effect or reflecting its recognition. Analyzing the best-cited publications can show which research topics have got the greatest attention from the scientific community [75]. Those interested in becoming experts in nutrition and COVID-19 research should familiarize themselves with the top-cited publications. The most cited article was by Di Renzo et al*.* [14] and published in the *Journal of Translational Medicine*. This Italian study was conducted to characterize the modifications that occurred in the lifestyles and eating behaviors of Italians during the COVID-19 pandemic. Approximately half of the sample perceived the concept of weight gain. Few participants stopped smoking and increased their physical activities. However, more than 38 percent committed to Mediterranean foods, especially in the age category of 18–30 years.

The second most cited article was by Ammar et al*.* [2] and was published in *Nutrients.* This international study, which included patients from all over the world, outlined that house arrest had a negative impact on people’s lifestyles during the COVID-19 pandemic, which included, but was not limited to, reducing all types of physical activity and increasing the consumption of unhealthy diets. The third most cited article was by Barazzoni et al*.* [44] and was published in *Clinical Nutrition.* In this study, the European Society for Clinical Nutrition and Metabolism (ESPEN) provided ten instructions to properly help physicians manage diet status in patients with coronavirus infection, particularly the elderly, patients with multimorbidity and those who require an intensive care unit. The fourth most cited article was by Calder et al. [11] and was published in *Nutrients.* This article summarized that there are enough essential vitamins and minerals to strengthen the immune system, which is necessary to combat COVID-19. It also concluded that these supplements are cheap, efficient, and without harm.

The fifth most cited article was by Galanakis [5] and published in Foods. This study focused on the challenges of food systems during the COVID-19 pandemic; creating a new system to reduce the cost of foods and make them sustainable. In addition, the researchers said that food should be provided with healthy nutritious substances that maintain immune function. The sixth most cited article was by Hobbs [45] and published in the *Canadian Journal of Agricultural Economics.* This study participated to talk about the challenges facing the supply chain system during the COVID-19 crisis. Importantly, the authors are advised to keep this system flexible and implement a plan to prevent damage of this model. At the same time, it has to maintain the supply of food for the highly susceptible population (i.e., poor people) and avoid stock shortages. The seventh most cited article was by Sidor and was Rzymski [15] and published in *Nutrients*. In this article, the authors reported on the impact of the COVID-19 pandemic on eating habits. They found that people who were overweight or obese were more likely to have bad habits, such as increased food consumption, gain weight, and low commitment to healthy diets (i.e., fruits).

The eighth most cited article was by Butler and Barrientos [10] and published in *Brain Behavior and Immunity*. This article highlighted the effects of nutritional habits on the vulnerability of COVID-19 and the outcomes that would occur in the long run. Clearly, Western pattern nutrition, which consists of saturated lipids, sweets, and processed carbohydrates, can negatively affect the immune function and weaken its ability to invade viruses, such as coronavirus. The ninth most cited article was by Muscogiuri et al. [17] and published in the *European Journal of Clinical Nutrition*. This study concluded that individuals should implement numerous tactics in their daily lifestyle and eating habits during the COVID-19 lockdown, aiming to avoid unhealthy diet behaviors (i.e., managing the amount and time of eating and snaking). The tenth most cited article was by Zabetakis et al. [12] and was published in *Nutrients*. A section of this article focused on the diet style that people should follow during the COVID-19 pandemic to maintain a healthy immune system and likely prevent infection. Individuals should choose foods rich in vitamins and healthy ingredients, such as Mediterranean foods, and avoid a Western diet pattern.

### Strengths and limitations

Unlike previous systematic analyzes and reviews, this is the first study to quantitatively and intuitively evaluate scientific papers on nutrition of COVID-19 published during the early phases of the pandemic and as such, it will be a useful reference for academics in this field. However, there were some limitations because we opted to limit the nutrition search to the titles of the publications rather than the abstracts. The search query was created to retrieve all potentially relevant papers in the field of nutrition and COVID-19. However, there is a risk of false positive or false-negative findings exists. Otherwise, the sample may include less representative publications that are not specifically about COVID-19 and nutrition. Furthermore, the bibliometric analysis in the current study is limited to nutrition- and COVID-19-related publications found in the Scopus database. Although Scopus is often regarded the most comprehensive and accurate database of publications and citations, it may not contain the entire collection of COVID-19 research. Other databases, such as PubMed, Web of Science, and Google Scholar, might have offered additional insights that were not available in this investigation. Furthermore, the number of citations and the number of publications will fluctuate over time due to the short period following the start of the pandemic and the ever-changing presence of COVID-19 research. In addition to that, numerous recently published, high-quality articles were unable to garner enough citations to be included in the list of the top ten most cited papers list. As a result, publications published in the most recent year (that is, 2021) were not included in this list, although this does not imply that those articles are less significant. Lastly, because the online Scopus database is constantly updated, there is some variation between our bibliometric results and the real findings. In this regard, new studies are still being published and a number of new studies are likely to appear in the coming months.

## Conclusions

This bibliographic study gives a general overview of publications related to nutrition and covid-19. Most publications were conducted in the USA and Italy, and Nutrients being the most popular source of articles. This study sheds light on the growth and development of nutrition and covid-19-related research and should contribute to the expansion of the global frontier in the major hot topics, including “food security in the COVID-19 pandemic”; “nutritional determinants and COVID-19 outcomes”; and “changes in diet habits during the COVID-19 pandemic and its consequences”. Understanding the growth of developing scientific knowledge on COVID-19 nutrition research, for example, is useful not only for the scientific community but also for evidence-based policymakers and nutritionists at the global level to improve the efficiency of future studies and better understand the role of diet and nutrition in COVID-19 to properly address the consequences of the COVID-19 pandemic's consequences.

## Data Availability

The data sets generated and/or analyzed during the current study are available upon request from the corresponding author (saedzyoud@yahoo.com).

## Abbreviations

Ifs: Impact factors
COVID-19: Coronavirus disease 2019
SDGs: Sustainable development goals
MeSH: Medical subject headings
